# Fatty Acid-Binding Protein 3 is Critical for α-Synuclein Uptake and MPP^+^-Induced Mitochondrial Dysfunction in Cultured Dopaminergic Neurons

**DOI:** 10.3390/ijms20215358

**Published:** 2019-10-28

**Authors:** Ichiro Kawahata, Luc Bousset, Ronald Melki, Kohji Fukunaga

**Affiliations:** 1 Department of Pharmacology, Graduate School of Pharmaceutical Sciences, Tohoku University, Sendai 980-8578, Japan; kawahata@tohoku.ac.jp; 2 CEA, Institut François Jacob (MIRcen) and CNRS, Laboratory of Neurodegenerative Diseases, 18 Route du Panorama, 92265 Fontenay-aux-Roses, France; Luc.BOUSSET@cnrs.fr (L.B.); ronald.melki@cnrs.fr (R.M.)

**Keywords:** fatty acid-binding protein 3, α-Synuclein, 1-methyl-4-phenylpyridinium (MPP^+^), mitochondria, synucleinopathy, Parkinson’s disease

## Abstract

α-Synuclein is an abundant neuronal protein that accumulates in insoluble inclusions in Parkinson′s disease and other synucleinopathies. Fatty acids partially regulate α-Synuclein accumulation, and mesencephalic dopaminergic neurons highly express fatty acid-binding protein 3 (FABP3). We previously demonstrated that FABP3 knockout mice show decreased α-Synuclein oligomerization and neuronal degeneration of tyrosine hydroxylase (TH)-positive neurons *in vivo*. In this study, we newly investigated the importance of FABP3 in α-Synuclein uptake, 1-methyl-4-phenylpyridinium (MPP^+^)-induced axodendritic retraction, and mitochondrial dysfunction. To disclose the issues, we employed cultured mesencephalic neurons derived from wild type or FABP3^−/−^ C57BL6 mice and performed immunocytochemical analysis. We demonstrated that TH^+^ neurons from FABP3^+/+^ mice take up α-Synuclein monomers while FABP3^−/−^ TH^+^ neurons do not. The formation of filamentous α-Synuclein inclusions following treatment with MPP^+^ was observed only in FABP3^+/+^, and not in FABP3^−/−^ neurons. Notably, detailed morphological analysis revealed that FABP^−/−^ neurons did not exhibit MPP^+^-induced axodendritic retraction. Moreover, FABP3 was also critical for MPP^+^-induced reduction of mitochondrial activity and the production of reactive oxygen species. These data indicate that FABP3 is critical for α-Synuclein uptake in dopaminergic neurons, thereby preventing synucleinopathies, including Parkinson′s disease.

## 1. Introduction

Parkinson′s disease (PD) [1] is a common neurodegenerative movement disorder which affects approximately 1% of people aged > 60 years [2,3]. Neuropathological features of PD include neuronal cell loss in the midbrain substantia nigra and the presence of cytoplasmic protein aggregates known as Lewy bodies (LBs) and Lewy neurites [4]. LBs contain various proteins [5] including tyrosine hydroxylase (TH), a rate-liming enzyme of dopamine [6]. α-Synuclein, a 140-amino acid protein associated with synaptic vesicles in presynaptic terminals [7], is the major filamentous component of LBs and Lewy neurites in PD and dementia with Lewy bodies (DLB) [8,9].

Accumulation of α-Synuclein and the presence of filamentous inclusions are hallmarks of the pathogenesis of PD and dementia with Lewy bodies [8,9]. We recently demonstrated a relationship between the uptake of α-Synuclein assemblies and the appearance of pathological hallmarks of synucleinopathies [10,11,12]. α-Synuclein toxicity is triggered when it undergoes oligomerization *in vitro* [13] and *in vivo* [14]. Therefore, the aggregation of α-Synuclein monomers is believed to play a crucial role in PD pathology. Recent studies revealed that the interaction between monomeric α-Synuclein and fatty acids or other monomers/oligomers accelerates the formation of α-Synuclein assemblies [15,16]. α-Synuclein oligomerization and its toxicity is dependent on the concentration of the monomers [17]. Furthermore, α-Synuclein uptake by dopaminergic neurons is indispensable and may play a role in synucleinopathies [18,19].

Various molecular mechanisms are expected for the uptake of α-Synuclein, for example, associating with α3-subunit of Na^+^/K^+^-ATPase (NKA) [20], neurexin [21,22], and particular endocytic pathways [23]. Previous reports also suggested that α-Synuclein binds to fatty acids, particularly long-chain polyunsaturated fatty acids [24,25,26]. α-Synuclein interaction with fatty acids accelerates its oligomerization [25]. Furthermore, FABP3 protein has been shown to promote 1-methyl-4-phenyl-1,2,3,6-tetrahydropyridine (MPTP)-induced α-Synuclein oligomerization [27], and the inhibition of FABP3 by its targeted compounds has been demonstrated to alleviate MPTP-induced oligomerization [28,29]. Altogether, these data suggest that FABP3 participates not only in α-Synuclein multimerization but also in the uptake of extracellular α-Synuclein and/or the turnover of the protein, which may favor oligomerization.

In this study, using PD model dopaminergic neurons [30], we demonstrated that FABP3 is critical for α-Synuclein uptake and that knocking out FABP3 completely abolished the fibrillization of α-Synuclein. In addition, we showed that FABP3 is also critical for MPP^+^-induced neurite retraction and the reduction of mitochondrial activity, which is accompanied by reactive oxygen species (ROS) formation.

## 2. Results

### 2.1. FABP3 is Critical for α-Synuclein Uptake in Cultured Mesencephalic Neurons

**Figure 1:**
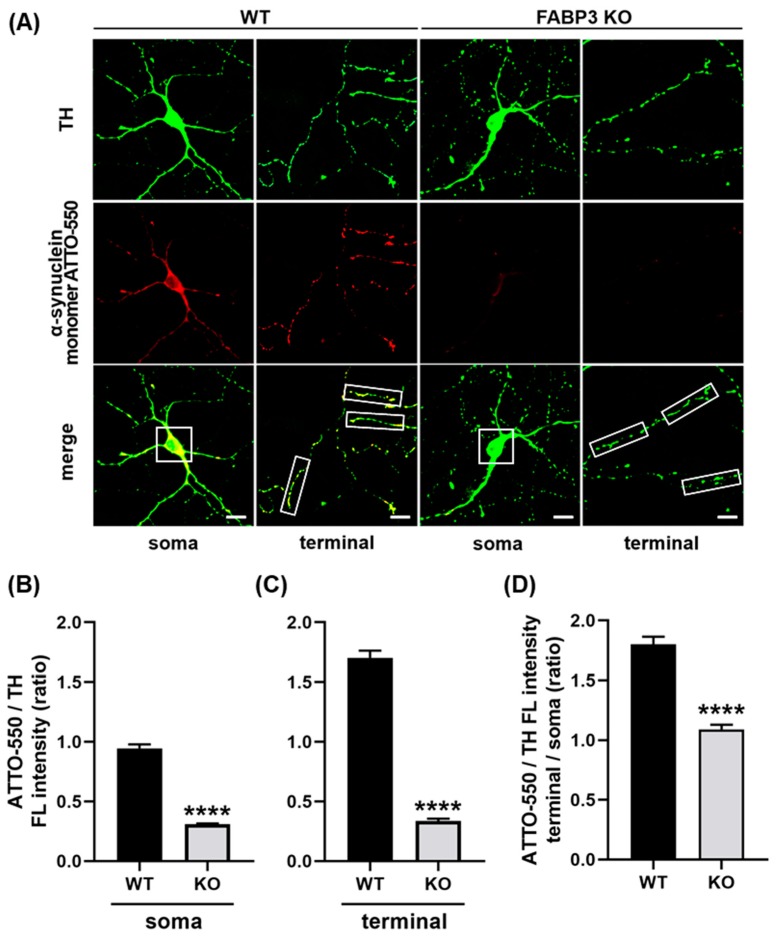
Cultured primary dopaminergic neurons require fatty acid-binding protein 3 (FABP3) to take up α-Synuclein. (**A**) Representative images of TH^+^ mesencephalic neurons at days *in vitro* (DIV) 12 derived from wild type (WT) or FABP3^−/−^ C57BL6 mice. Neurons were exposed to 1 μM ATTO-550-labeled α-Synuclein monomer for 48 h and stained with antibody against tyrosine hydroxylase (TH, green). Scale bar: 10 μm. (**B**) Quantitative analysis of the ratio of ATTO-550-labeled α-Synuclein fluorescence intensity (FL) to TH immunoreactivity in the region of interest (ROI)-selected soma of individual TH^+^ neurons shown in A (white square 15 × 15 μm). **** *p* < 0.0001 in wild type (WT) versus FABP3^−/−^ (KO), *n* > 20. (**C**) Ratio of ATTO-550 fluorescence intensity to TH immunoreactivity in ROI-selected neuronal processes shown in A (white square 10 × 30 μm). **** *p* < 0.0001 in WT versus KO, *n* > 60. (**D**) The calculated ratio of ATTO-550 to TH in the individual terminal (C) was divided by the value in the soma (B) to represent the superiority on the α-Synuclein uptake in the axonal processes compared to the uptake in the soma. **** *p* < 0.0001 in WT versus KO, *n* > 20.

To investigate whether FABP3 is required for α-Synuclein uptake, we prepared cultured mesencephalic neurons derived from wild type or FABP3^−/−^ C57BL6 mice and exposed them to 1 μM ATTO-550-labeled α-Synuclein monomer for 48 h. In this experiment, we measured the fluorescent intensity of the uptaken ATTO-550-labeled α-Synuclein monomers in FABP3^+/+^ or FABP3^−/−^ TH^+^ dopaminergic neurons. We found that FABP3^+/+^ TH^+^ neurons take up ATTO-labeled α-Synuclein and showed intracellular accumulation of the protein (Figure 1A). In contrast, the internalization of α-Synuclein was dramatically attenuated in the FABP3^−/−^ TH^+^ cells (Figure 1A,B, **** *p* < 0.0001). Detailed quantification analysis revealed that the intensity of ATTO in neurites was higher than that in the soma (Figure 1B,C, **** *p* < 0.0001) suggesting that α-Synuclein uptake is greater in neurites than in cell bodies. In FABP3^−/−^ TH^+^ neurons, the ATTO fluorescent ratio of neurites to soma decreased (Figure 1D, **** *p* < 0.0001 in terminal/soma ratio), which implied that knocking out FABP3 preferentially impairs α-Synuclein uptake at neuronal processes and terminals.

### 2.2. FABP3 Deficiency Abolishes MPP^+^-Induced Formation of α-Synuclein Inclusions in Cultured Mesencephalic Neurons

**Figure 2:**
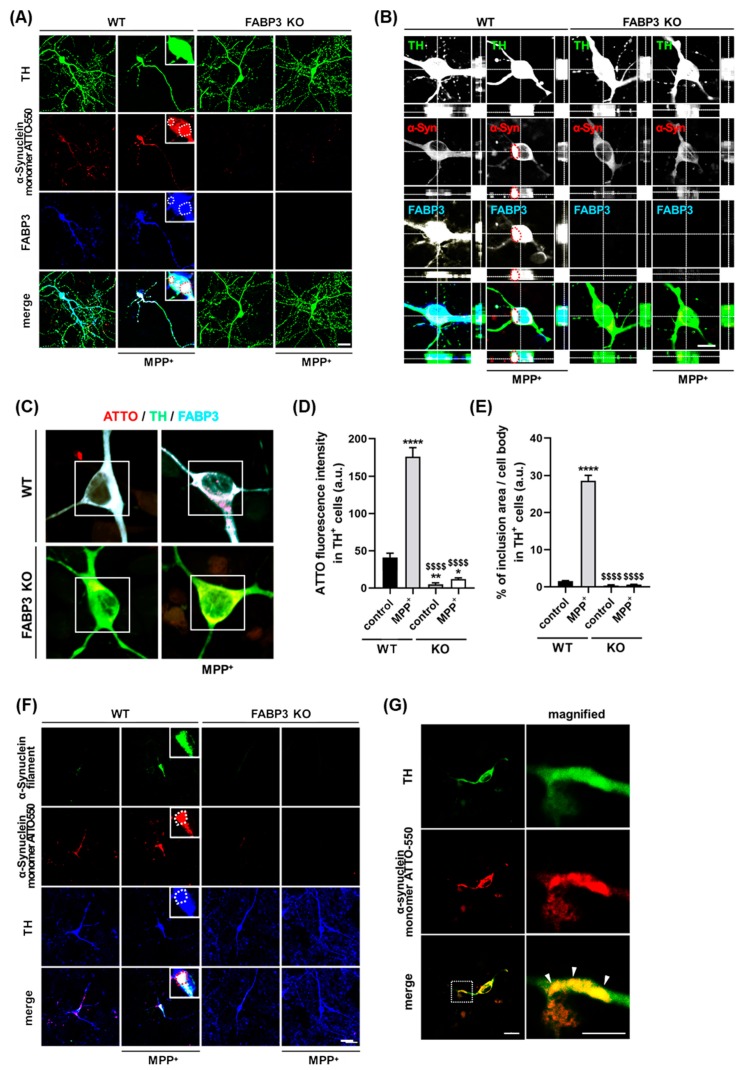
FABP3 is required for the formation of α-Synuclein inclusions in 1-methyl-4-phenylpyridinium (MPP^+^)-treated dopaminergic neurons. (**A**) Representative images of TH^+^ mesencephalic neurons at DIV 12 derived from wild type (WT) and FABP3^−/−^ C57BL6 mice. Neurons were exposed to 1 μM ATTO-550-labeled α-Synuclein monomer with or without 10 μM MPP^+^ for 48 h. The cells are stained with antibodies against TH (green) and FABP3 (blue). The dotted circles indicate α-Synuclein and FABP3-positive aggregates. Scale bar: 20 μm. (**B**) Confocal microscopy reveals ATTO-labelled α-Synuclein within the intracellular compartment of dopaminergic neurons. Image taken from an optical section approximately in the center of the z-axis of a dopaminergic neuron stained with anti-TH and anti-FABP3 antibodies. The red dotted circles indicate α-Synuclein and FABP3-positive aggregates. Scale bar: 10 μm. (**C**) Representative images of TH^+^ mesencephalic neurons either treated or not treated with MPP^+^. The images were acquired in photon counting mode using Leica TCS SP8 with a higher magnification (×63 objective). Scale bar: 10 μm. (**D**) Quantitative analysis of ATTO-550-labeled α-Synuclein monomer fluorescence intensity in ROI-selected soma of individual TH^+^ neurons shown in C (white square). **** *p* < 0.0001, ** *p* < 0.01, * *p* < 0.05 versus WT control; ^$$$$^ *p* < 0.0001 versus WT MPP^+^; *n* > 60. (**E**) Percentage of ATTO-550-labeled α-Synuclein-positive inclusions (defined as dot-like signals 3 standard deviations (SD) above the background levels) in the total cell area [31]. **** *p* < 0.0001 versus WT control; ^$$$$^ *p* < 0.0001 versus WT MPP^+^; *n* > 60. (**F**) Representative images of TH^+^ mesencephalic neurons in the same treatment conditions as the neurons in A. The cells are stained with antibodies against α-Synuclein filament (green) and TH (blue). Scale bar: 20 μm. (**G**) Representative images of a TH^+^ mesencephalic neuronal process stained with an antibody against TH (green). The magnified images of the dotted box are shown in the right, and the white arrowheads indicate Lewy neurite-like structures. Scale bar: 10 μm.

We next investigated whether FABP3 is critical for the MPP^+^-induced formation of α-Synuclein inclusions in cultured mesencephalic neurons. To analyze FABP3 dependency in the MPP^+^-induced aggregation of α-Synuclein, we exposed cultured neurons from either C57BL6 wild type FABP^+/+^ (WT) mice or FABP3 knockout FABP3^−/−^ (KO) mice to 1 μM ATTO-550-labeled α-Synuclein monomer with or without 10 μM MPP^+^ at days *in vitro* (DIV) 10. We show here that the ATTO-550-labeled α-Synuclein monomer formed inclusions after 48 h of treatment with MPP^+^ in FABP3^+/+^ neurons (Figure 2A, WT). In contrast, ATTO-α-Synuclein aggregations were completely abolished in TH^+^ neurons of FABP3^−/−^ background (Figure 2A, KO). Furthermore, confocal images along the z-axis revealed ATTO-α-Synuclein inclusions in TH-positive neurons co-localizing with FABP3 in WT mice, whereas no ATTO-positive inclusions were observed in FABP3 KO mice (Figure 2B). FABP3 knockout was confirmed by immunostaining using anti-FABP3 antibody (Figure 2A,B, blue).

To further characterize the dependence of α-Synuclein inclusion formation on FABP3, we quantified the fluorescence intensity of ATTO-550 α-Synuclein in the cell bodies of TH^+^ neurons (Figure 2C). MPP^+^ treatment accelerated the accumulation of α-Synuclein in the soma, which was dramatically decreased in FABP3^−/−^ neurons (Figure 2D, ^$$$$^ *p* < 0.0001 versus WT MPP^+^). Importantly, knocking out FABP3 greatly decreased the area of α-Synuclein inclusions (Figure 2E, ^$$$$^ *p* < 0.0001 versus WT MPP^+^), suggesting that FABP3 participates not only in the uptake but also in the coalescence of α-Synuclein within neurons. We further investigated whether ATTO-positive α-Synuclein inclusions are of fibrillar nature considering that the process of fibrillization is important to the formation of Lewy bodies in PD [32]. Analysis using anti-filamentous α-Synuclein-specific antibody revealed that ATTO-positive inclusions (Figure 2F, red) are co-localized with α-Synuclein filament immunoreactivity in FABP3^+/+^, but not in FABP3^−/−^ neurons (Figure 2F, green), indicating that the exposure of FABP3^+/+^ dopaminergic neurons to MPP^+^ triggered ATTO-550-labeled α-Synuclein fibrillization. α-Synuclein-positive inclusions, apparently similar to the Lewy neurites, were also observed in the neuronal processes (Figure 2G).

### 2.3. FABP3 is Critical for MPP^+^-Induced Loss of Neurites in Dopaminergic Neurons

**Figure 3:**
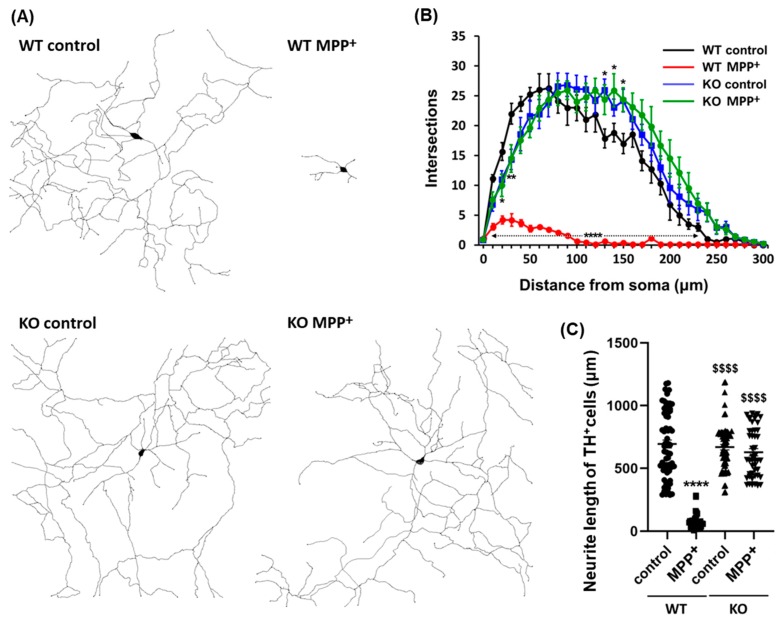
Representative dendritic morphology of TH^+^ dopaminergic neurons derived from wild type or FABP3^−/^^−^ mice. (**A**) Representative traced dendritic patterns of wild type (WT) or FABP3^−/^^−^ (KO) dopaminergic neurons either treated or not treated with 10 μM MPP^+^ for 48 h. (**B**) Sholl analysis of WT or FABP3 KO dopaminergic neurons either treated or not treated with MPP^+^. **** *p* < 0.0001, ** *p* < 0.01, * *p* < 0.05 versus WT control, *n* > 20. (**C**) Average neurite length of WT or FABP3^−/^^−^ dopaminergic neurons. **** *p* < 0.0001 versus WT control; ^$$$$^ *p* < 0.0001 versus WT MPP^+^, *n* > 20.

Alterations in neurite morphology, including loss of axodendritic processes and changes in branching patterns, are hallmarks of Parkinson′s disease and cell models of the disease. Herein, we investigated whether FABP3 participates in the development of morphological characteristics in MPP^+^-treated dopaminergic neurons. Interestingly, FABP3 knockout neurons exhibited a unique resistance to MPP^+^-induced neurite retraction (Figure 3A). The exposure of TH^+^ dopaminergic neurons from WT mice to 10 μM MPP^+^ for 48 h led to neurite retraction (Figure 3A, WT MPP^+^). In contrast, no significant retraction of axodendritic processes was observed in TH^+^ dopaminergic neurons from FABP3 knockout mice upon exposure to MPP^+^ (Figure 3A, KO MPP^+^).

We also analyzed the number of neurite branches in order to determine the morphological characteristics of FABP3^+/+^ and FABP3^−/−^ neurons. Sholl analysis revealed that MPP^+^ treatment led to the loss of dendritic complexity in the TH^+^ neurons from WT mice but not in those from FABP3 knockout mice (Figure 3B). Knocking out FABP3 neither drastically altered the morphological features of dopaminergic neurons, although the number of branches increased at a distance of 130–150 μm away from the cell body (Figure 3B, * *p* < 0.05 versus WT control), nor affected the total neurite length compared to WT dopaminergic neurons (Figure 3C). The decrease in neurite length following exposure of WT dopaminergic neurons to MPP^+^ was not observed in FABP3^−/−^ dopaminergic neurons (Figure 3C, ^$$$$^ *p* < 0.0001 versus WT MPP^+^). These data suggest that FABP3 plays a key role in the process of MPP^+^-induced neuronal degeneration. 

### 2.4. FABP3 is Essential for MPP^+^-Induced Mitochondrial Dysfunction in Dopaminergic Neurons

**Figure 4:**
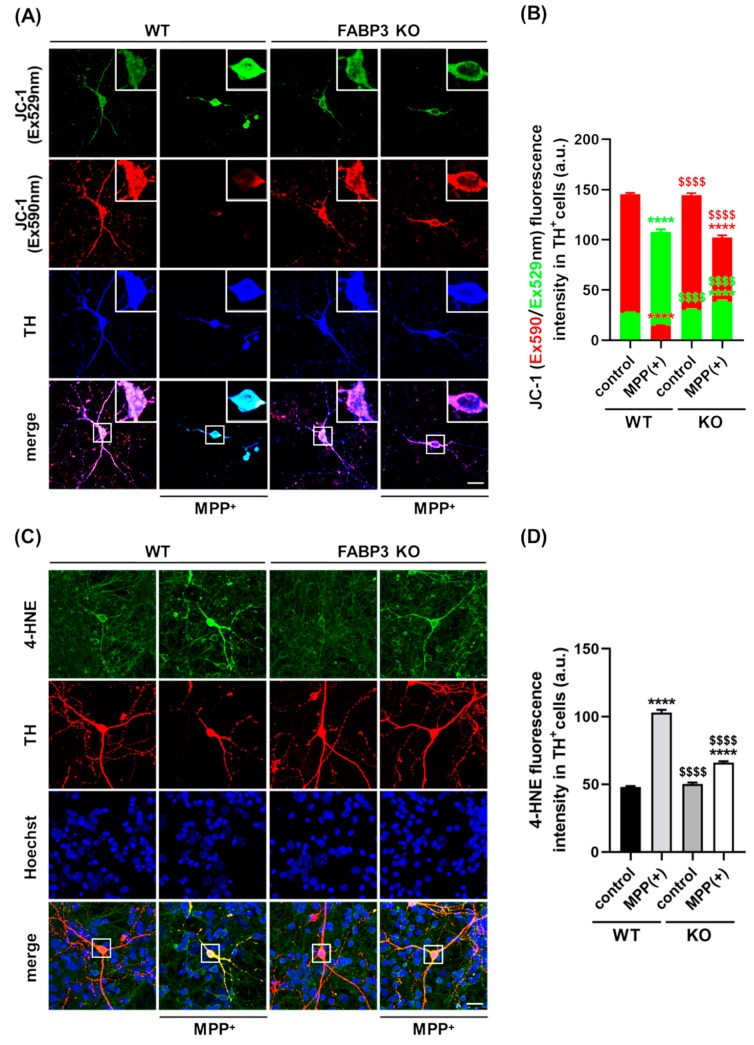
FABP3 is involved in MPP^+^-induced reduction of mitochondrial activity and ROS production in dopaminergic neurons. (**A**) Representative images of JC-1-stained TH^+^ neurons. The white boxes indicate regions of interest (ROIs), which are magnified in the upper right in each image and quantified to measure the fluorescence intensity. Scale bar: 10 μm. (**B**) Quantitative analysis of Ex590 (red) and Ex529 (green) fluorescence intensity in JC-1-stained TH^+^ neurons. **** *p* < 0.0001 versus wild type (WT) control; ^$$$$^ *p* < 0.0001 versus WT MPP^+^, *n* > 20. (**C**) Representative images of TH^+^ neurons (red) stained with anti-4HNE antibody (green). The white boxes show ROIs to measure 4-HNE fluorescence intensity. Scale bar: 10 μm. (**D**) Quantitative analysis of 4-HNE immunoreactivity in TH^+^ cells. **** *p* < 0.0001 versus WT control; ^$$$$^ *p* < 0.0001 versus WT MPP^+^, *n* > 20.

The mitochondria play a central role in aging-related neurodegenerative diseases such as PD [30,33] and is required to maintain neuronal processes [34]. Additionally, evidence of the loss of mitochondrial integrity following α-Synuclein aggregation has been reported [10]. To determine whether FABP3 plays a role in mitochondrial dysfunction-associated axodendritic retraction, we assessed its involvement in mitochondrial activity and reactive oxygen species (ROS) generation. To document the role played by FABP3 in the maintenance of mitochondrial integrity, FABP^+/+^ and FABP3^−/−^ TH^+^ neurons were subjected to MPP^+^ treatment, and the loss of the mitochondrial membrane potential (ΔΨm) was measured. To this end, dopaminergic neurons were incubated with the cationic dye JC-1, which exhibits specific mitochondrial localization and produces red fluorescence in vital cells if ΔΨm is intact, as well as produces green fluorescence upon loss of ΔΨm. In WT control, confocal microscopy revealed a red-dotted staining pattern, indicating the accumulation of JC-1 in the active mitochondria (Figure 4A). Upon MPP^+^ treatment, TH^+^ neurons showed an increase in cytosolic green fluorescence and a decrease in red fluorescence (Figure 4A,B). Quantitative analysis of the JC-1 red/green fluorescence intensity ratio revealed a loss of ΔΨm in MPP^+^-treated TH^+^ dopaminergic neurons in both FABP^+/+^ and FABP3^−/−^ neurons (Figure 4B, **** *p* < 0.0001 versus WT control in red columns). However, intriguingly, FABP3^−/−^ dramatically attenuated MPP^+^-induced loss of ΔΨm in TH^+^ neurons (Figure 4B, ^$$$$^ *p* < 0.0001 versus WT MPP^+^ in red columns). These data indicate that FABP3 is critical for MPP^+^-induced reduction of mitochondrial activity.

We further investigated cytosolic ROS production following MPP^+^ treatment by immunocytochemistry using a specific antibody for 4-hydroxy-2-nonenal (4-HNE), a sensitive marker of oxidative damage and lipid peroxidation [35]. Treatment with 10 μM MPP^+^ for 48 h elevated 4-HNE levels in both FABP3^+/+^ and FABP3^−/−^ neurons (Figure 4C,D, **** *p* < 0.0001 versus WT control). However, the level of MPP^+^-induced 4-HNE in FABP3 knockout neurons was significantly lower than those in WT neurons (Figure 4D, ^$$$$^ *p* < 0.0001 versus WT MPP^+^). These data suggest that FABP3 is involved in MPP^+^-induced ROS production. MPP^+^ is taken up by the dopamine transporter (DAT) in dopaminergic neurons, which results in selective degeneration [36]; therefore, we also tested DAT expression in dopaminergic neurons. FABP3^−/−^ neurons showed no significant alteration in the immunofluorescence level of anti-DAT antibody in TH^+^ neurons (Supplementary Figure S1), suggesting that knocking out FABP3 does not directly affect the level of DAT gene expression.

## 3. Discussion

α-Synuclein, whether aggregated or not, has been shown to traffic and propagate *in vitro* and *in vivo.* It is widely accepted that this process might contribute to Parkinson’s disease pathology. The role of FABP3 in this process is unclear. To address this issue, we assessed the uptake of exogenous ATTO-550-labeled α-Synuclein in primary mesencephalic neurons derived from WT or FABP3^−/−^ C57BL6 mice. To our knowledge, our study is the first to demonstrate the involvement and importance of FABP3 in α-Synuclein uptake. Our data provide new insights into processes that accelerate the formation of α-Synuclein inclusions through the interaction of exogenous monomeric α-Synuclein with endogenous fatty acids and oligomeric α-Synuclein [15,16].

We demonstrated that FABP3 is key for extracellular α-Synuclein monomer uptake using cultured dopaminergic neurons. Indeed, the uptake of ATTO-labeled α-Synuclein monomer is attenuated in FABP3^−/−^ neuronal cells, in particular in their processes (Figure 1). MPP^+^ induced intracellular aggregation of ATTO-labeled α-Synuclein (Figure 2) in FABP3^+/+^ neurons, which have filamentous shapes (Figure 2E, **** *p* < 0.0001 versus WT control; and Figure 2F), but not in FABP3^−/−^ neurons (Figure 2E, no significance versus WT control; ^$$$$^ *p* < 0.0001 versus WT MPP^+^; and Figure 2F). This suggests that FABP3 is essential for the aggregation of α-Synuclein to form filamentous-shaped inclusions in dopaminergic neurons.

We also showed that FABP3 plays a central role in MPP^+^-induced axodendritic retraction. The treatment of FABP3^+/+^, but not FABP3^−/−^, neurons with MPP^+^ (Figure 3A,C, **** *p* < 0.0001 versus WT control) resulted in the loss of neuronal processes. The number of intersections assessed by Sholl analysis revealed that FABP3 is required for the MPP^+^-induced loss of neuronal branching in TH^+^ neurons (Figure 3B). Intriguingly, even though the total neurite length was not altered, increased intersections at distances of 130, 140, and 150 μm from the soma was observed in FABP3^−/−^ neurons (*p* < 0.05 versus WT control, Figure 3B,C). Previous studies showed that the expression of FABP is increased in the brains of patients suffering from schizophrenia and autism [37] and that the dendritic spine density is reduced in schizophrenia patients [38]. Although the distinct role of FABP3 in the generation of neuronal processes is still unclear, the increased neuronal branching in FABP3^−/−^ neurons, which we reported in our study, indicated that FABP3 negatively modulates axonal generation and maturation in dopaminergic neurons.

Retraction of neuronal processes often results from mitochondrial dysfunction [34]. Analysis of the mitochondrial membrane potential using JC-1 staining revealed that MPP^+^ increased the intensity of red fluorescence (590 nm) and decreased the intensity of green fluorescence (529 nm) in both FABP^+/+^ and FABP3^−/−^ dopaminergic neurons (Figure 4A,B, **** *p* < 0.0001 versus WT control), indicating that MPP^+^ is effective in both FABP^+/+^ and FABP3^−/−^ neurons. However, the extent of MPP^+^-induced ΔΨm reduction significantly differs in FABP^+/+^ and FABP3^−/−^ neurons (Figure 4A,B, ^$$$$^ *p* < 0.0001 versus WT control in red columns). These data suggest that FABP3 is involved in the MPP^+^-induced ΔΨm reduction in dopaminergic neurons. Furthermore, analysis of 4-HNE fluorescence intensity, which is a marker of oxidative stress during fatty acid oxidation, revealed that FABP3 accelerates MPP^+^-induced ROS production (Figure 4C,D, ^$$$$^ *p* < 0.0001 versus WT MPP^+^). Thus, FABP3 may play a role in the MPP^+^-induced mitochondrial dysfunction and ROS generation in dopaminergic neurons.

We previously demonstrated that the α3-subunit of Na^+^/K^+^-ATPase (NKA) interacts with extracellular α-Synuclein [20]. Dysfunction of the neuron-specific α3-subunit is associated with rapid-onset dystonia Parkinsonism (RDP) and alternating hemiplegia of childhood (AHC). Thus, we tested whether FABP3 affects NKA expression in the somatodendritic regions of TH^+^ neurons. As shown in Supplementary Figure S2, α3-NKA levels in the cell bodies and terminals did not significantly change (Supplementary Figure S2). We further explored the mechanism of FABP3-dependent α-Synuclein uptake by examining the effect of FABP3 knockout on somatodendritic neurexin expression in TH^+^ neurons since α-Synuclein interacts with neurexin [21,22]. Although we examined the effect of knocking out FABP3 on the somatodendritic expression of neurexin, quantitative analysis revealed no significant alteration in neurexin immunoreactivities (Supplementary Figure S3). An inhibitor of micropinocytosis, cytochalasin D [23], also showed no significant alteration in the fluorescence intensity of ATTO-550-labeled α-Synuclein monomers in FABP^−/−^ TH^+^ neurons (data not shown). This indicates that a loss of uptake of α-Synuclein due to FABP3 knockout is not sensitive to cytochalasin D. These data suggest that FABP3-dependent uptake of α-Synuclein is regulated by a novel, unique mechanism which has not yet been identified. We will continue to further explore the molecular mechanism of FABP3-dependent uptake of α-Synuclein and the associated-caveola constituent proteins.

Altogether, our observations provide new insights into the function of FABP3 in neuronal cells. FABP3 appears to be critical for the uptake of extracellular α-Synuclein monomers and its aggregation into intracellular filamentous-shaped inclusions in cultured mesencephalic neurons by a novel unidentified mechanism. FABP3 plays a role in MPP^+^-induced neuronal retraction, mitochondrial activity, and oxidative stress. Our data suggest that FABP3 contributes to the vulnerability of dopaminergic neurons during neuronal degeneration in Parkinson’s disease and other synucleinopathies.

## 4. Materials and Methods

### 4.1. Animals

Pregnant C57BL/6J wild type mice were purchased from Japan SLC (Shizuoka, Japan). FABP3^−/−^ mice [39,40] were housed under climate-controlled conditions with a 12-h light and dark cycle. Animal studies were conducted in accordance with the Tohoku University institutional guidelines. Ethical approval (Number 2017PhL-004 approved on 29 March 2018 and valid until 28 March 2023) was obtained from the Institutional Animal Care and Use Committee of the Tohoku University Environmental and Safety Committee.

### 4.2. Cell Culture

Primary cultures of mesencephalic neurons were prepared as previously described [41]. The dissected tissues were then treated with papain (Sumitomo bakelite, Tokyo, Japan) and mechanically dissociated into single-cell suspensions. The cells were plated out onto poly-L-lysine-coated cover glass chambers at a density of 3 × 10^5^ cells/cm^2^. The cultures were maintained in Eagle’s minimum essential medium (FUJIFILM Wako Pure Chemical, Osaka, Japan) supplemented with 10% fetal calf serum for 1–4 DIV or 10% horse serum for 5–12 DIV. The cells were cultured at 37 °C in an atmosphere of 5% CO_2_ in the air with 100% relative humidity. All animal experiments were conducted in accordance with the general guidelines for animal experiments at Tohoku University.

### 4.3. Reagents

ATTO-550-labeled α-Synuclein monomer was prepared as previously described [12]. 1-methyl-4-phenylpyridinium (MPP^+^), the active metabolite of the neurotoxin 1-methyl-4-phenyl-1,2,5,6-tetrahydropyridine (MPTP), was purchased from Sigma and prepared as 10 mM stock in dimethyl sulfoxide (DMSO, FUJIFILM Wako Pure Chemical). Cultured neurons were either treated or not treated with 1 μM ATTO-550-labeled α-Synuclein monomer and 10 μM MPP^+^ for 48 h. To examine the sensitivity of FABP3-dependent uptake of α-Synuclein to cytochalasin D, which is an inhibitor of pinocytosis [23], we exposed cultured mesencephalic neurons to 2 μM cytochalasin D (Merck, Sigma-Aldrich, St. Louis, MO, USA).

### 4.4. Immunocytochemistry

Cultured mesencephalic neurons were fixed for immunocytochemistry at 12 DIV. Following fixation with 4% paraformaldehyde for 30 min, they were incubated with 0.1% Triton X-100 for 15 min. After pre-blocking with 5% goat serum in PBS for 1 h, they were then incubated overnight at 4 °C with the following primary antibodies: Rabbit anti-TH affinity-purified polyclonal antibody (1:400; Millipore, AB152, Billerica, MA, USA), mouse anti-human FABP3 monoclonal antibody, clone 66E2 (1:50; Hycult Biotech, HM2016, Uden, Netherlands), rabbit anti-α-Synuclein filament conformation-specific polyclonal antibody (1:5000, Abcam, ab209538, Cambridge, United Kingdom). To visualize Na^+^/K^+^-ATPase and neurexin-1, we also applied anti-ATP1A3 (α3-subunit of Na^+^/K^+^-ATPase) mouse monoclonal antibody (1:200; Abcam, ab2826) and anti-neurexin-1-β mouse monoclonal antibody (1:50; Merck, Sigma-Aldrich, MABN607). After washing with phosphate-buffered saline (PBS), the cells were incubated with either Alexa Fluor 405-, 488-, or 546- conjugated secondary antibodies (1:500 dilution, Invitrogen, Carlsbad, CA, USA). Mitochondrial activity was visualized using the JC-1 MitoMP Detection Kit (Dojindo, MT09, Kumamoto, Japan). Images were acquired by confocal laser scanning microscope (TCS SP8, Leica Microsystems, Wetzlar, Germany). For acquiring higher magnification images, we used the photon counting mode of the Leica HyD scanner (Leica Microsystems) (Figure 2C).

### 4.5. Analyses of Fluorescence Intensity and Morphological Characteristics

Quantitative analysis of fluorescence intensity was performed using NIH ImageJ 1.52 software as previously described [31]. First, the background signal intensities were measured from regions without any cells and subtracted from all the images, and the remaining signals of cells were used to define total cell areas.

To define the morphological characteristics of cultured mesencephalic neuronal processes, individual TH-positive neurons were randomly selected and analyzed by Sholl analysis [42,43]. Sholl analysis was performed using the Sholl tool of Fiji J [44] to quantify the number of intersections at 10 μm intervals from the soma [45]. Statistical analysis was performed by comparing the number of intersections of the cultured mesencephalic neurons derived from FABP3^−/−^ mice with those from cultured mesencephalic neurons from wild type mice for each 10 μm interval using GraphPad Prism 8 (GraphPad Software, CA, USA).

### 4.6. Statistical Analyses

All values are expressed as mean ± standard error of mean (SEM). Statistical significance was tested by one-way analysis of variance (ANOVA) with post-hoc Tukey’s multiple comparison test or two-way ANOVA with post-hoc Bonferroni’s multiple comparison test. A *p* value < 0.05 was considered as statistically significant. All the statistical analyses were performed using GraphPad Prism 8 (GraphPad Software, CA, USA).

## Supplementary Materials

Supplementary materials can be found at https://www.mdpi.com/1422-0067/20/21/5358/s1.

## Abbreviations

FABP3	Fatty acid-binding protein 3
TH	Tyrosine hydroxylase
MPP^+^	1-Methyl-4-phenylpyridinium
4-HNE	4-Hydroxynonenal
DAT	Dopamine transporter
PD	Parkinson’s disease
DLB	Dementia with Lewy bodies
NKA	Na^+^/ K^+^-ATPase
PBS	Phosphate-buffered saline
SEM	Standard error of mean
DIV	Days *in vitro*
