# Supplementary material for: Fatty Acid-Binding Protein 3 is Critical for α-Synuclein Uptake and MPP+-Induced Mitochondrial Dysfunction in Cultured Dopaminergic Neurons
Source: Int J Mol Sci. 2019 Oct 28;20(21):5358. doi: 10.3390/ijms20215358 (PMC6862506; doi:10.3390/ijms20215358)
Supplement: Supplementary file 1 [file ijms-20-05358-s001.pdf]

Supplementary Materials

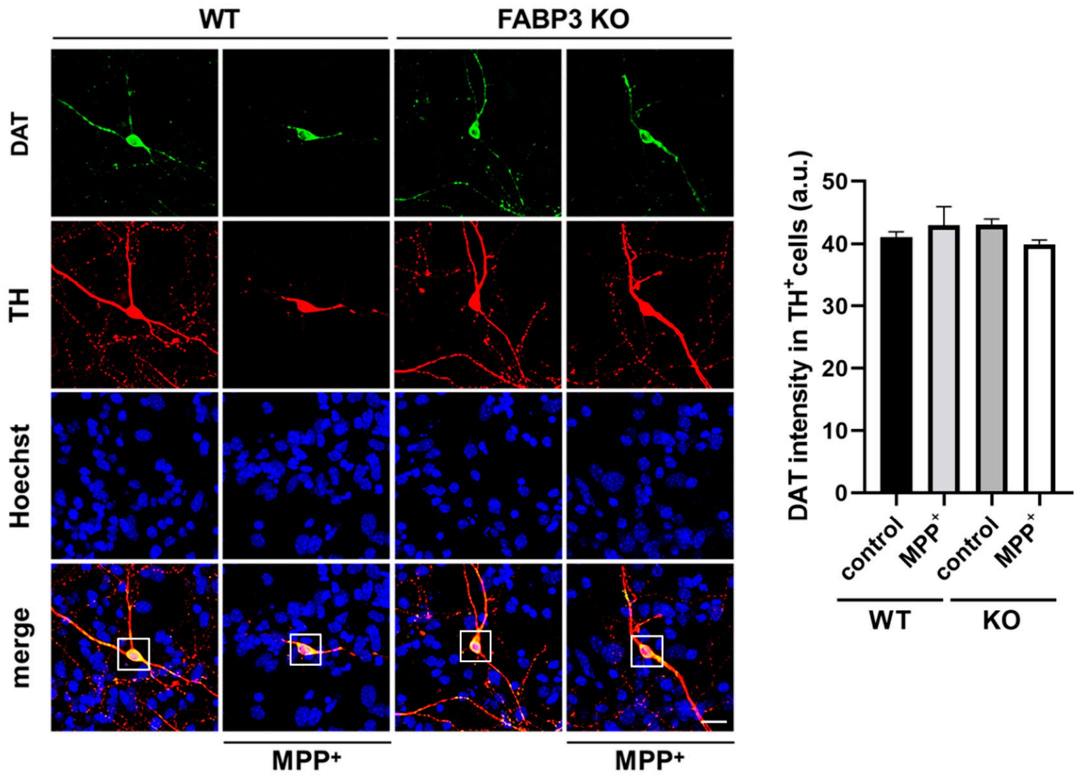

Figure S1. Knocking out FABP3 does not directly affect the levels of dopamine transporter (DAT) gene expression in cultured mesencephalic neurons. Scale bar: 20 μm. Right columns show quantitative analysis of DAT immunoreactivity in TH^+ cells. No significance was observed, $n > 20$.

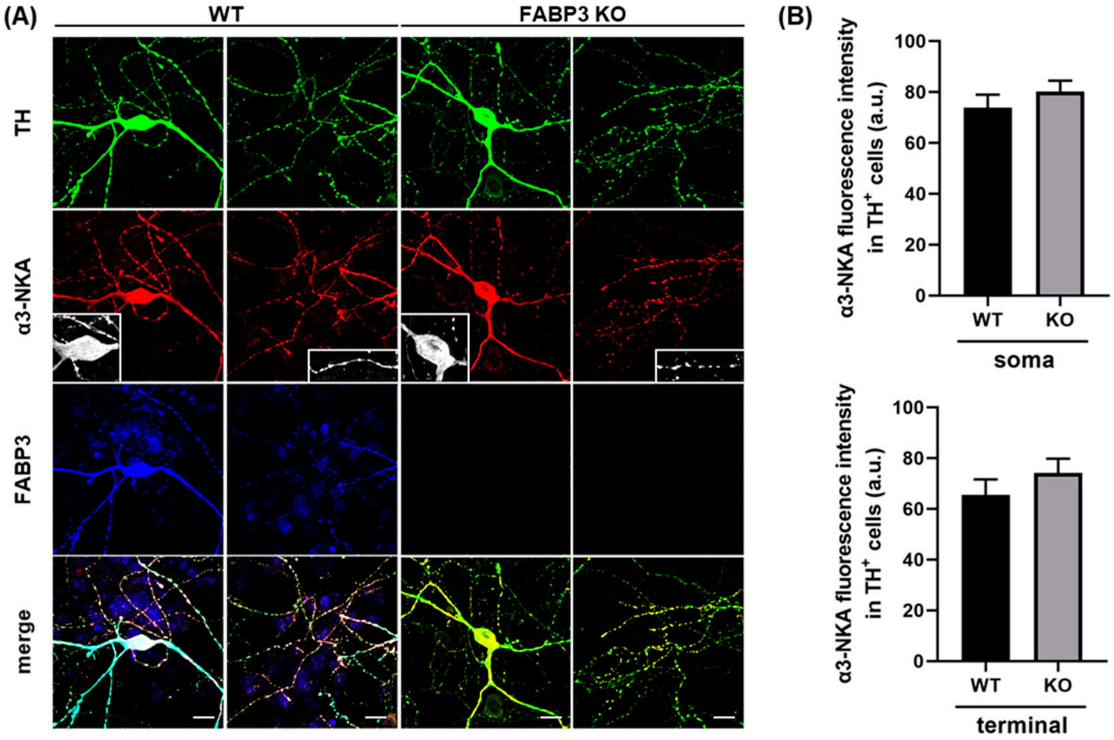

Figure S2. Knocking out FABP3 does not directly affect the levels of Na+/K+-ATPase (NKA) α3-subunit gene expression in cultured mesencephalic neurons. (**A**) Representative images of α3-NKA immunoreactivities (red) in TH^+ neurons. Scale bar: 10 μm. (**B**) Columns show quantitative analysis of α3-NKA immunoreactivity in the cell bodies (upper) and in the terminals (lower) of TH^+ cells. No significance was observed, $n > 20$.

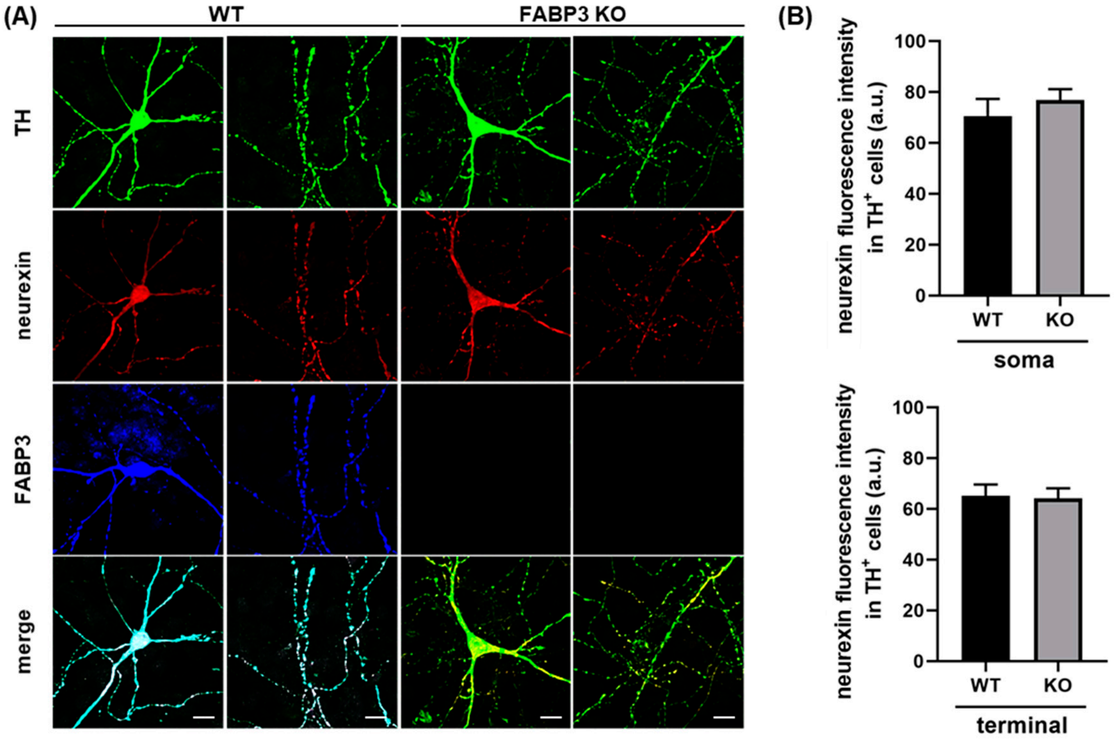

Figure S3. Knocking out FABP3 does not directly affect the levels of the neurexin gene expression in cultured mesencephalic neurons. (**A**) Representative images of neurexin immunoreactivities (red) in TH^+ neurons. Scale bar: 10 μm. (**B**) Columns show quantitative analysis of neurexin immunoreactivity in the cell bodies (upper) and in the terminals (lower) of TH^+ cells. No significance was observed, $n > 20$.
